# Immune evasion activities of accessory proteins Vpu, Nef and Vif are conserved in acute and chronic HIV-1 infection

**DOI:** 10.1016/j.virol.2015.03.015

**Published:** 2015-08

**Authors:** Petra Mlcochova, Luis Apolonia, Silvia F. Kluge, Aishwarya Sridharan, Frank Kirchhoff, Michael H. Malim, Daniel Sauter, Ravindra K. Gupta

**Affiliations:** a Department of Infection, University College London, London, UK; b Department of Infectious Diseases, King׳s College London, London, UK; c Institute of Molecular Virology, Ulm University Medical Center, Ulm, Germany

**Keywords:** HIV, Vpu, Nef, Vif, Accessory, Acute, Chronic

## Abstract

Heterosexual HIV-1 transmission has been identified as a genetic bottleneck and a single transmitted/founder (T/F) variant with reduced sensitivity to type I interferon initiates productive infection in most cases. We hypothesized that particularly active accessory protein(s) may confer T/F viruses with a selective advantage in establishing HIV infection. Thus, we tested *vpu*, *vif* and *nef* alleles from six T/F and six chronic (CC) viruses in assays for 9 immune evasion activities involving the counteraction of interferon-stimulated genes and modulation of ligands known to activate innate immune cells. All functions were highly conserved with no significant differences between T/F and CC viruses, suggesting that these accessory protein functions are important throughout the course of infection.

## Highlights

• Transmitted/founder viruses are known to be relatively interferon resistant.
• Transmitted/founder HIV-1 viruses have fully functional accessory genes Vpu, Vif and Nef.
• Vpu, Vif and Nef functions do not appear to differ between acute and chronic infection.

## Introduction

Studies have defined a genetic ‘bottleneck’ at mucosal HIV-1 transmission with a single inferred variant initiating peripheral viremia in most cases (Baalwa et al., 2013; Fenton-May et al., 2013; Haaland et al., 2009; Ochsenbauer et al., 2012; Parrish et al., 2013; Salazar-Gonzalez et al., 2009). In particular it has been noted that transmitted founder (T/F) Envelope proteins are R5 tropic with both increased sensitivity to broadly neutralizing antibodies (Wilen et al., 2011) and reduced ability to interact with maraviroc-bound CCR5 compared to viruses isolated from chronic infection (Parker et al., 2013). Furthermore, preferential transmission of ancestral as opposed to contemporary strains in donor individuals has been reported (Redd et al., 2012), suggesting specific viral determinants for sexual transmission that differ from determinants for propagation of chronic infection. Intriguingly, it has been reported that T/F viruses are less restricted by interferon (IFN) in primary T cells than chronic controls (Fenton-May et al., 2013; Parrish et al., 2013).

Lentiviral accessory proteins have evolved the ability to mitigate the detrimental effects of an IFN-induced antiviral immune response by interfering with various host immunity factors (Harris et al., 2012; Moll et al., 2010; Shah et al., 2010). For example, HIV-1 Vpu and Vif counteract the IFN-inducible restriction factors tetherin/BST-2/CD317 (Neil et al., 2008; Van Damme et al., 2008) and APOBEC3D/F/G, respectively (Bishop et al., 2004; Hultquist et al., 2011; Sheehy et al., 2002; Zheng et al., 2004). Vpu also prevents expression of IFN and IFN-stimulated genes by inhibiting the activation of NF-κB (Bour et al., 2001; Sauter et al., 2015), and Nef is known to modulate a range of host immune receptors including CD4 and MHC molecules (Kirchhoff, 2010).

We hypothesized that accessory gene(s) from T/F viruses confer advantages to establishment and early propagation of HIV-1 infection either through counteraction of IFN-stimulated genes or modulation of ligands known to activate NK, NKT and other immune cells. We therefore directly compared functional activities of the accessory proteins Vpu, Vif and Nef from six T/F and six CC viruses (see Table 1 for patient characteristics and Supplementary Figs. 1–3 for multiple sequence alignments).

## Results

### Counteraction of tetherin/BST-2/CD317 and NF-κB activation by Vpu

**Fig. 1:**
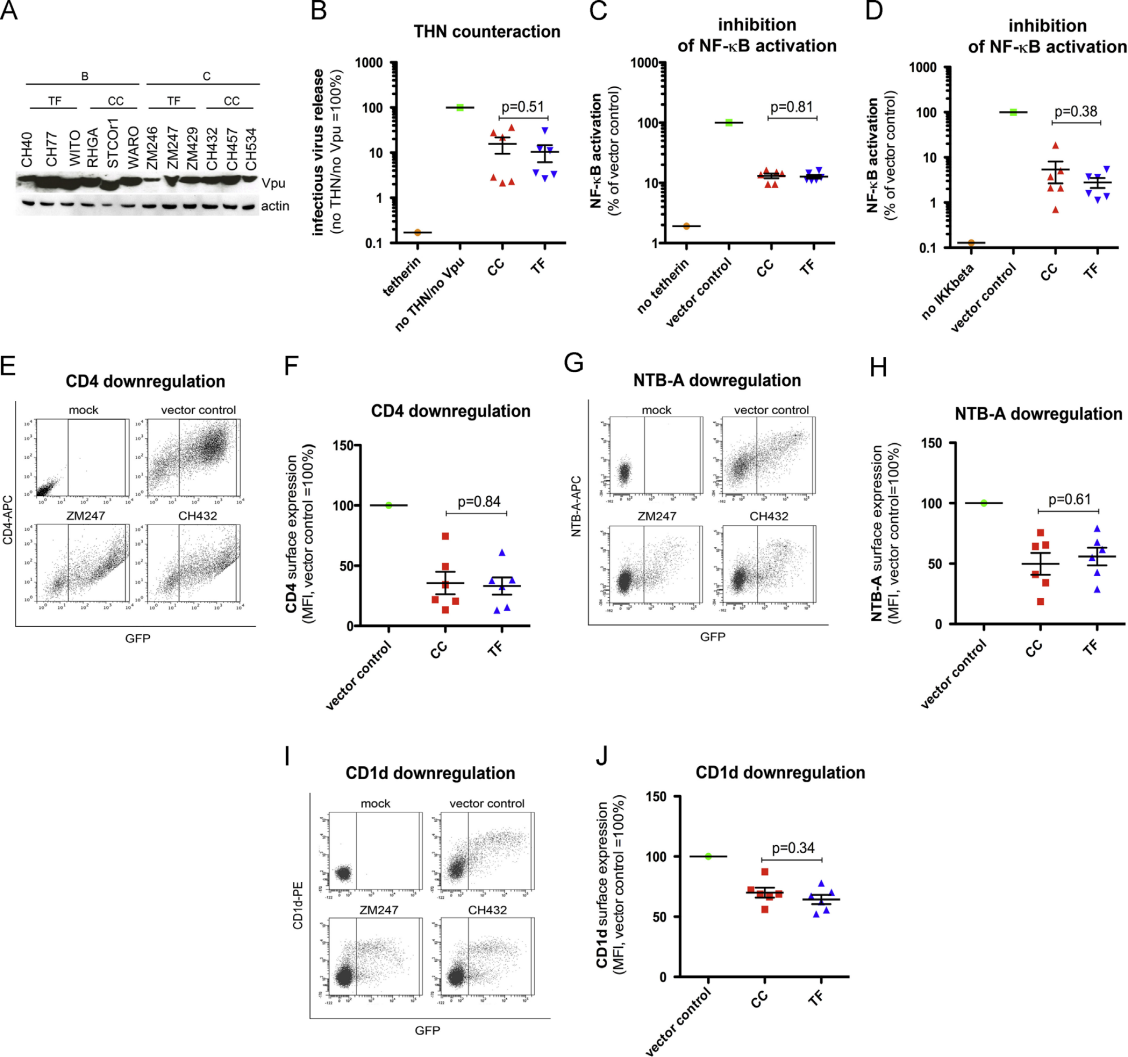
Vpu-mediated counteraction of tetherin, inhibition of NF-κB activation and downmodulation of CD4, NTB-A and CD1d. (A) Immunoblot showing expression of T/F and CC Vpu proteins in HEK293T cells transfected with pCG *vpu* IRES eGFP expression constructs. An antibody against AU-1 was used to detect C-terminally tagged Vpu. (B) HEK293T cells were co-transfected with expression vectors for HIV-1 Gag-Pol, VSV-G, a modified HIV genome encoding firefly luciferase, Vpu and tetherin or an empty vector control. 48 h post-transfection, viral supernatants were used to infect TZM-bl cells and luciferase activity was measured 24 h post-infection. Infectious virus yield in the presence of Vpu was normalized to that in the presence of empty vector (vector control). (C and D) HEK293T cells were co-transfected with Vpu expression plasmids, along with plasmid expressing an NF-κB-dependent firefly luciferase reporter construct and a pTAL promoter *gaussia* luciferase plasmid for normalization, and either a plasmid expressing (C) human tetherin or (D) a constitutively active mutant of IKKβ. 40 h post- transfection, dual luciferase assays were performed. Firefly luciferase signals were divided by the corresponding *gaussia* luciferase signals and normalized to the vector control. **(E–J)** HEK293T cells were transfected with (E and F) CD4 (G and H) NTB-A or (I and J) CD1d expression vectors and plasmids expressing eGFP alone or together with Vpu. Two days post-transfection CD4, NTB-A or CD1d surface expression was detected by fluorophore-conjugated antibodies and examined by flow cytometry. Mean fluorescence intensities were normalized to the control construct expressing only eGFP (vector control). Each data point represents one *vpu* allele. The mean of three independent experiments ± sd is shown. In all experiments, unpaired Student׳s *t* test was performed to examine differences between T/F and CC viruses. (E,G and I) Examples of primary experimental data from flow cytometry for one T/F and CC Vpu.

Vpu proteins from T/F and CC viruses were tested for their ability to increase virion release by counteracting tetherin/BST-2/CD317. Tetherin serves as a physical tether between viral and cellular membranes and inhibits the egress of budding virions from infected cells. All Vpus were expressed (Fig. 1A) and enabled efficient virus release from tetherin expressing HEK293T cells. Anti-tetherin activity was similar for Vpus from T/F and CC viruses (*p*=0.51, Fig. 1B), consistent with a previous study demonstrating conservation of tetherin antagonism over extended periods in chronically infected patients (Pickering et al., 2014).

Tetherin does not only restrict virion release but also acts as an innate sensor activating NF-κB-dependent expression of type I IFN and IFN-stimulated genes (Galao et al., 2012). We therefore investigated whether T/F Vpus are more efficient in blocking tetherin signaling than Vpus from CC viruses. A dual luciferase reporter assay in HEK293T cells revealed that all Vpus efficiently inhibited the activation of NF-κB with no significant differences between the T/F and CC groups (*p*=0.81, Fig. 1C). Since Vpu has also been reported to block activation of NF-κB independently of tetherin (Bour et al., 2001; Sauter et al., 2015), we repeated the experiment using a constitutively active mutant of IKKβ instead of tetherin as the inducer. Again, T/F and CC Vpus suppressed NF-κB activation to similar degrees (*p*=0.31, Fig. 1D).

### Cell surface downregulation of CD4, NTB-A and CD1d by Vpu

Besides counteracting tetherin and inhibiting NF-κB activation, Vpu also decreases cell surface expression of CD4, NTB-A and CD1d. While downmodulation of CD4 prevents superinfection and enables the release of fully infectious progeny virions from infected cells (Kimura et al., 1994; Lama et al., 1999; Wildum et al., 2006), downmodulation of NTB-A and CD1d has been suggested to protect HIV-1 infected cells from NK or NKT cell mediated killing, respectively (Moll et al., 2010; Shah et al., 2010). Using flow cytometry, we monitored expression of these receptors on the surface of HEK293T cells transfected with Vpu or a vector control. Vpu reduced surface levels of all three receptors as expected, without significant differences between T/F and CC viruses (CD4: *p*=0.84, NTB-A: *p*=0.61, CD1d: *p*=0.34, Fig. 1E–J).

### Counteraction of APOBEC3F/G by Vif

**Fig. 2:**
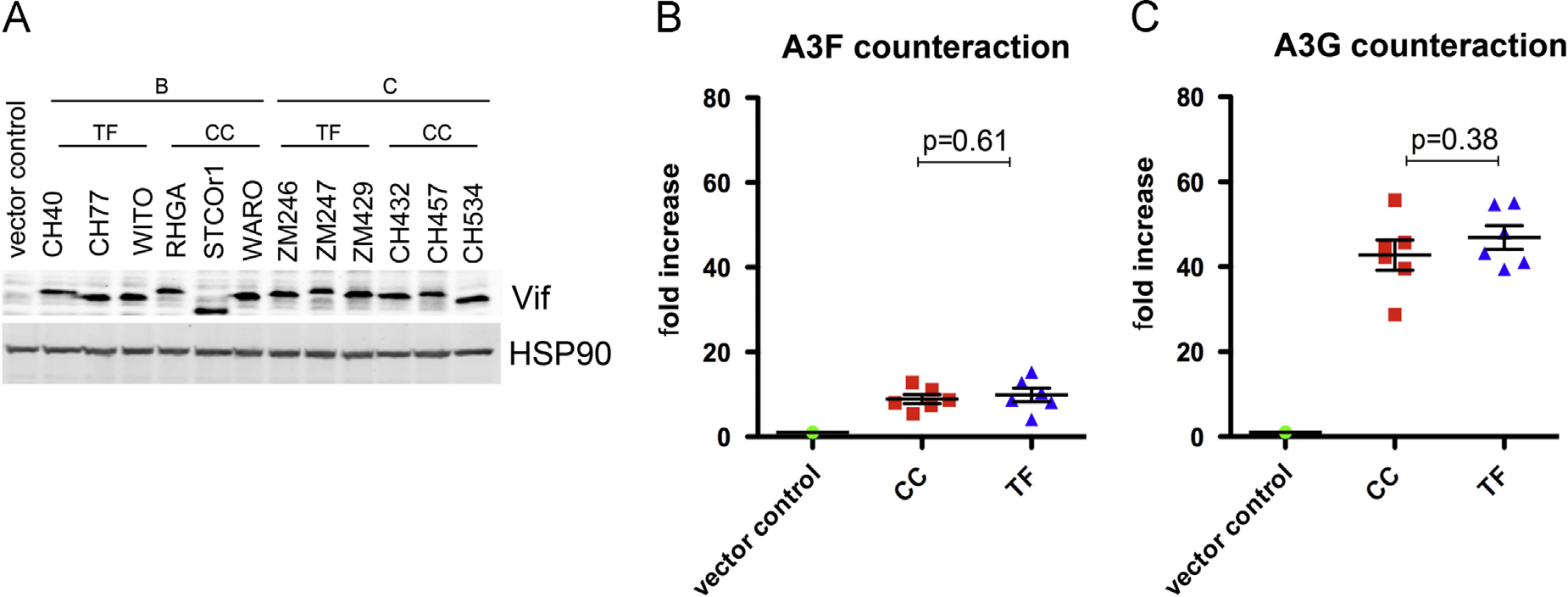
Vif-mediated counteraction of APOBEC3F and APOBEC3G. HEK293T cells were transfected with plasmids encoding NL4-3 Δ*vif*, APOBEC3F or G and HA-tagged Vif proteins. 48 h post-transfection, producer cells and supernatants were harvested. (A) Expression of Vif proteins in producer cells was assessed by standard immunoblot. (B and C) Virion infectivity of supernatants generated in the presence of APOBEC3F (B) or APOBEC3G (C) was determined using TZM-bl indicator cells. Luciferase values were normalized to controls without Vif (vector control). The mean of four independent replicates ±sd is shown.

The accessory protein Vif induces the ubiquitination and subsequent degradation of the restriction factors APOBEC3DF/G/H to prevent their incorporation into viral particles (Bishop et al., 2004; Hultquist et al., 2011; Sheehy et al., 2002). APOBEC3F/G are cytidine deaminases, inhibiting HIV-1 replication through G to A hypermutation of viral cDNA as well as direct inhibition of reverse transcription itself (Gillick et al., 2013). We analyzed the activity of T/F and CC Vif proteins against human APOBEC3F and APOBEC3G by co-transfecting HEK293T cells with plasmids encoding *vif*-deficient HIV-1 along with APOBEC3F or APOBEC3G and *vif* alleles/empty plasmid. As Vif expression/ stability varied between alleles tested, the amount of Vif expression plasmid was modified in order to achieve similar amounts of Vif protein in the producer cells (Fig. 2A). Under these conditions the infectivity of supernatant viruses was determined, revealing that Vifs from T/F and CC viruses have equivalent activity against APOBEC3F and APOBEC3G (Fig. 2B and C).

### Cell surface downregulation of CD4, CD28 and MHC class I by Nef

**Fig. 3:**
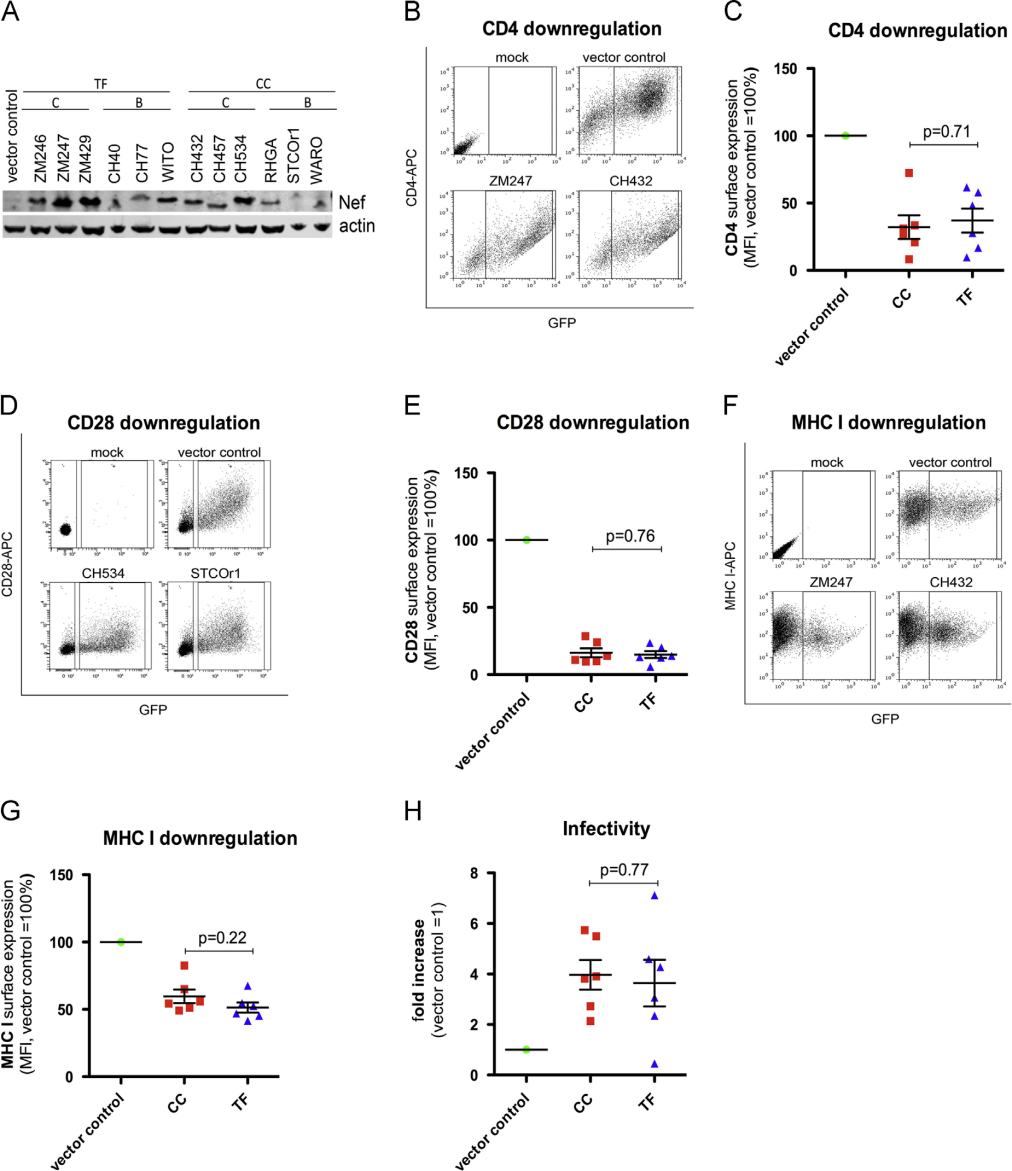
Nef-mediated downmodulation of CD4, CD28, MHC class I and enhancement of virion infectivity. (A) Immunoblot showing expression of T/F and CC Nef proteins in HEK293T cells transfected with pCG_*nef* IRES eGFP expression constructs. An antibody against AU-1 was used to detect C-terminally tagged Nef. (B–E) HEK293T cells were transfected with (B and C) CD4 or (D and E) CD28 expression vector and constructs expressing eGFP alone or together with Nef. Two days post-transfection CD4 or CD28 expression was detected by flow cytometry. Mean fluorescence intensities were normalized to the control construct expressing only eGFP (vector control). (F and G) MHC class I downregulation using HeLa.B27 cells transfected with constructs expressing eGFP alone or together with Nef. Two days post-transfection MHC I expression was analyzed by flow cytometry as described above. (H) Virion infectivity enhancement by Nef was measured using HEK293T cells co-transfected with HIV-1 NL4-3 and eGFP Nef. Supernatants were harvested 48 h post-transfection and p24-normalized virus was used to infect TZM-bl cells. Infectivity was normalized to the control construct expressing no *nef* allele (vector control). The mean of at least three independent experiments ±sd is shown. (B,D and F) Example of primary experimental data from flow cytometry for one T/F and CC Nef.

The accessory protein Nef downregulates various cell surface proteins including CD4, MHC class I and CD28 to escape immune surveillance (Garcia and Miller, 1991; Schwartz et al., 1996; Swigut et al., 2001) and may thus also be involved in the reduced sensitivity of T/F viruses to IFN. CD4 and CD28 downregulation were analyzed by transient transfection of HEK293T cells with vectors expressing Nef and either CD4 or CD28. All Nef proteins were efficiently expressed (Fig. 3A) and reduced surface expression of both receptors with no significant differences between T/F and CC viruses (CD4: *p*=0.71, CD28: *p*=0.76, Fig. 3B–E). To explore MHC class I downregulation, a HeLa cell line expressing HLA-B^⁎^27 was transfected with different *nef* alleles and MHC class I cell surface expression was analyzed by FACS 48 h post-transfection. Nef proteins of both groups of viruses downmodulated MHC class I to a similar extent (*p*=0.22, Fig. 3F and G).

### Nef-mediated enhancement of virion infectivity

It has been shown that HIV-1 virion infectivity is reduced in the absence of a functional *nef* gene (Aiken and Trono, 1995; Chowers et al., 1994). We thus tested the effect of Nef proteins on virion infectivity by transfecting HEK293T cells with *nef* alleles and *nef*-deficient HIV-1 NL4-3. Supernatants harvested two days post-transfection were used to infect indicator TZM-bl cells with normalized amounts of p24. Despite variation between individual Nef proteins in their ability to enhance virion infectivity, there was no statistical difference when T/F and CC Nefs were compared (*p*=0.77, Fig. 3H).

## Discussion

In summary, our results show that established functions of the accessory proteins Vpu, Vif and Nef are conserved across T/F and CC viruses. Specifically, our results from transient expression studies suggest that the increased IFN resistance of newly transmitted HIV-1 strains is not the result of a particularly efficient Vpu-mediated inhibition of NF-κB activation or counteraction of the restriction factors tetherin and APOBEC3F/G. An important limitation is that viral proteins were not expressed from a provirus in our experiments, and therefore it is possible that *in vivo* expression may differ.

The identification of traits that are shared by T/F viruses may ultimately provide novel targets for strategies aiming to prevent the acquisition of HIV-1 at mucosal surfaces. Thus, further analyses of accessory viral gene function in primary human cells and systematic comparison of other HIV-1 gene products are highly warranted to decipher the reason(s) for the increased IFN resistance and selection advantage of T/F viruses during transmission and acute infection.

## Materials and methods

### Cell culture and transfections

HEK293T, TZM-bl and HeLa.B27 cells were grown under standard conditions in Dulbecco׳s Modified Eagle Medium (DMEM) supplemented with 10% fetal bovine serum, 120 mg/ml streptomycin, 120 mg/ml penicillin and 2 mM l-glutamine. Cells were transfected using the calcium-phosphate precipitation method, polyethyleneimine (PEI) or Fugene 6 (Roche Applied Science).

### Expression vectors

Vpu, Nef, CD1d, CD4, NTB-A (transcript variant 2) and BST-2/tetherin alleles were cloned into the CMV-promoter-based pCG expression vector, *vpu* and *nef* encoding vectors co-expressing eGFP or DsRed via an IRES as previously described (Sauter et al., 2009). CD28 was cloned into in a pcDNA3.1+ vector. HA-tagged subtype B and C Vif alleles were amplified from transmitted/founder and chronic full-length IMCs (infectious molecular clones) kindly provided by Beatrice Hahn and cloned into pcDNA3.1 using NotI and XhoI restriction sites. The plasmids encoding T7-A3F/G have been previously described (Apolonia et al., 2015). The firefly luciferase reporter plasmid containing three NF-κB binding sites as well as the constitutively active mutant of IKKβ were kindly provided by Bernd Baumann. A minimal promoter *gaussia* luciferase construct was purchased from Clontech (#631909) and used for normalization. It contains the TATA-like promoter (pTAL) region from the Herpes simplex virus thymidine kinase (HSV-TK) that is not responsive to NF-κB.

### Western blotting

Protein expression was assessed by standard immunoblots using anti AU-1 antibody (bs-2114R, Bioss) to detect AU1 tagged Vpu and Nef proteins or anti HA (3F10, Roche) to identify HA-tagged Vif. Anti-HSP90 (H114, Santa Cruz Biotechnology) and anti-actin antibody (AC-15, Abcam) were used to detect the loading control proteins.

### Flow cytometry

To determine the effect of Vpu on CD1d or NTB-A, HEK293T cells were transfected with 1 μg of the CD1d or NTB-A expression vector and 5 μg of pCG_IRES eGFP constructs expressing eGFP alone or together with Vpu. Two days post-transfection CD1d or NTB-A expression was examined by FACS analysis, essentially as described previously (Sauter et al., 2013). A phycoerythrin-conjugated anti-CD1d antibody (BD 550255) or an APC-conjugated anti-SLAM6 antibody (R&D FAB19081A) were used for staining. Fluorescence of stained cells was detected by two-color flow cytometry and Vpu-mediated down-modulation of the respective protein was calculated. Briefly, the mean fluorescence intensities were determined for cells showing specific ranges of eGFP expression. The fluorescence values obtained for cells transfected with the control construct expressing only eGFP was compared with the corresponding number obtained for cells coexpressing Vpu and eGFP to determine the efficiency of CD1d or NTB-A down-modulation.

To determine the effect of Vpu and Nef on CD4, HEK293T cells were transfected with 0.05 μg of the CD4 expression vector and 0.15 μg of pCG_IRES eGFP constructs expressing eGFP alone or together with Vpu or Nef. Two days post- transfection CD4 expression was examined by FACS analysis. A PE/Cy5 anti-human CD4 Antibody (BioLegend) was used for staining. Fluorescence of stained cells was detected by two-color flow cytometry and quantified as described above.

To determine the effect of Nef on CD28, HEK293T cells were transfected with 1 μg of the CD28 expression vector and 0.15 μg of pCG_IRES eGFP constructs expressing eGFP alone or together with Nef. Two days post-transfection CD28 expression was examined by FACS analysis. APC-conjugated anti-human CD28 antibody (CD28.2, BD Biosciences) was used for staining.

To determine the effect of Nef on MHC I, HeLa.B27 cells [a kind gift from Anthony Antoniou] were transfected with 0.5 μg of pCG_IRES eGFP constructs expressing eGFP alone or together with Nef. Two days post-transfection MHC I expression was examined by FACS analysis. A PE/Cy5 anti-human HLA-A,B,C antibody, clone W6/32 (BioLegend) was used for staining. Fluorescence of stained cells was detected by two-color flow cytometry and quantified as described above.

### NF-κB reporter assay

Transfections for luciferase assays were performed in 96-well plates and each transfection was performed in triplicate as described before (Sauter et al., 2013). Briefly, Vpu expression plasmids (100 ng) were transfected in HEK293T cells along with a plasmid expressing human tetherin or a constitutively active mutant of IKKβ (40 ng), an NF-κB-dependent firefly luciferase construct (100 ng) and a pTAL promoter *gaussia* luciferase reporter plasmid (25 ng). 40 h post-transfection, dual luciferase assays were performed. Firefly luciferase signals were normalized to the corresponding *gaussia* luciferase signals.

### Luciferase reporter virus assay

HEK293T cells were co-transfected with 0.3 μg of p8.91 HIV-1 gag-pol expression vector, 0.3 μg VSV-G, and 0.45 μg of pCSLUCW retroviral expression vector encoding luciferase. For tetherin counteraction experiments, 0.25 μg of T/F or CC Vpu constructs were co-transfected with 0.05 μg of tetherin, or empty vector (pcDNA3.1). 48 h post-transfection, viral supernatants were harvested, filtered using 0.45 µm pore-sized filters and used to infect TZM-bl cells. Luciferase activity of the TZM-bl cells was measured 24 h post-infection using the Steady Glo Firefly Luciferase assay (Promega) and GloMax96 Luminometer (Promega).

### Nef infectivity assay

HEK293T cells were cotransfected with 0.4 μg pBR_HIV-1 M NL4-3 (*nef* STOP) and 2 μg of pCG_Nef IRES eGFP in six well plates using the Calcium phosphate method. Virus was harvest from supernatant 48 h post-transfection and normalized using p24^Gag^ ELISA. Normalized virus was used to infect indicator TZM-bl cells and β-galactosidase activity measured 72 h post-infection using Gal-screen substrate (Applied Biosystems, USA).

### A3F/G restriction assay

HEK293T cells were co-transfected with *vif*-deficient NL4-3 (Phalora et al., 2012) and T7-A3F/G (Apolonia et al., 2015) at a 1:1 ratio. Cells were also co-transfected with plasmids encoding HA-tagged Vif proteins from T/F and CC viruses. 48 h after transfection, supernatants containing viruses were harvested, filtrated through a 45 μm pore-sized filter and quantified using a p24^Gag^ ELISA (Perkin Elmer). Viruses were then used to infect indicator TZM-bl cells and luciferase activity was measured 48 h post-infection. Producer cells were lysed in RIPA buffer (50 mM Tris–HCl pH 7.4, 100 mM NaCl, 1 mM MgCl_2_, 1% NP-40, 0.1% SDS and 0.5% sodium deoxycholate). Protein expression was assessed by standard immuno blot, anti HA (3F10, Roche) to identify HA-tagged Vif and anti-HSP90 (H114, Santa Cruz Biotechnology). Proteins were then detected by Li-cor Odyssey infrared imaging using IRDye800CW or IRDye 680LT-labeled secondary antibodies.

## Figures and Tables

**Table 1:** Origins and characteristics of viruses used in this study.

Virus	Country of origin	Gender	Transmission route	Fiebig stage	Viral load at isolation	Cd4 count at isolation	Coreceptor tropsim	TF/CC	HIV-1 subtype
ZM246F	Zambia	F	Heterosexual	II	10,013,800	NA	R5	TF	C
ZM247F	Zambia	F	Heterosexual	II	10,823,500	NA	R5	TF	C
ZM249M	Zambia	M	Heterosexual	IV	>2,000,000	NA	R5	TF	C
CH040	USA	M	MSM	II	2,197,248	NA	R5	TF	B
WITO	USA	M	Heterosexual	II	325, 064	NA	R5	TF	B
CH077	USA	M	MSM	II/III	394, 649	NA	R5/X4	TF	B
CH432	Malawi	M	Heterosexual	NA	40,570	261	R5	CC	C
CH457	Tanzania	F	Heterosexual	NA	234,671	450	R5	CC	C
CH534	South Africa	F	Heterosexual	NA	NA	NA	NA	CC	C
RHGA	USA	M	MSM	NA	50,000	571	R5	CC	B
WARO	USA	F	MSM	NA	16,758	598	R5	CC	B
STCOr1	USA	M	MSM	NA	67,964	796	R5/X4	CC	B

Key: MSM: men who have sex with men; TF – transmitted/founder virus; CC- Chronic virus; NA – not available; Feibig stage as described previously (Fiebig et al., 2003)

## Appendix A Supplementary materials

**Supplementary Figure 1:** amino acid alignment of Vpu alleles used in this study.

**Supplementary Figure 2:** amino acid alignment of Vif alleles used in this study

**Supplementary Figure 3:** amino acid alignment of Nef alleles used in this study

## Footnotes

Supplementary data associated with this article can be found in the online version at doi:10.1016/j.virol.2015.03.015.
