# Supplementary material for: Immune evasion activities of accessory proteins Vpu, Nef and Vif are conserved in acute and chronic HIV-1 infection
Source: Virology. 2015 Aug;482:72–8. doi: 10.1016/j.virol.2015.03.015 (PMC4503796; doi:10.1016/j.virol.2015.03.015)
Supplement: Supplementary file 1 — Supplementary Figure 1: amino acid alignment of Vpu alleles used in this study. [file mmc1.doc]

ZM246F MVDLLAKVDYRLGVGALIVALIIAIIVWTIVYIEYRKLLRQRKLDWLVKRVRERAEDSGNESDGDIEELSTMVDMEHIRLFD--DL

ZM247 MLEL----DYKIAIAALIVALIIAIVVWTIVYIEYRKLVRQRKIDWLIKRIRERAEDSGNESDGDQEELSTMVDMGHIRLLGAIDL

ZM249 MISLIEKVDYRLGVGALIVALIIAIVVWTIAYIEYRKLLRQRKIDWLIKRIRERAEDSGNESDGDTEELSTMVDMEHLRLLDVNEL

CH432 MIDLT-----ALGVAALIVALIIAIVVWTIAYIEYRKLVRQRKIDCLIKRIRERAEDSGNESEGDTEELSTLVDMGNLRLLDVNDL

CH457 MLDLLARVDYRIGVAALLIALIIAIVVWTIVYIEYKKLLRQKRIDWLIKRIRERAEDSGNESDGDIEEVETLVDMGNLRLLDVIN-

CH534 MLNL----DYRVGIGALIVALNIAIVVWIIVYIEYRKLVRQRRIDWLVKRIRERAEDSGNESDGDTEELSTMVDMGHLRLLDVIDL

CH40 MNSL-----QISAIVALVVAGIIAIVVWSIVAIEYRKILRQRKIDRLIDRIRERAEDSGNESDGDQEELSALVERGHLALGDINDL

CH77 MQSL-----NIA-IGALIVAAILAIIVWTIVFIEYRKILKQRKIDRLIERISERAEDSGNESDGDQEELSKLMEMGHHAPWDVNDL

WITO MQPL-----EILAVVALVVALILAIVVWTIVYIEYRKIQKQKKIDRLIDRIRERAEDSGNESDGDQEELSALVEMGHHAPWDVNDE

STCOr1 MQPL-----HIAAIVGLVVAAILAIVVWSIVFIEYKKIRRQNKIDRLIERISERAEDSGNESDGDQEELSALMEMGHHAPWDVNDL

RHGA MLPL-----NILAVAAFVVAAILAIIVWSIVFIEYRRILRQRKIDRLIDRIRDRAEDSGNESEGDQEELSELVERGHLAPWDVNDM

WARO MQSL-----EIIAIVALVVATIIAIVVWSIVLIEYRKILRQRKIDRIIDRIIERAEDSGNESEGDQEELSILVEMGHDAPWDINDL

* * : .:::* :**:** *. ***..: .*..:* ::.*: .*********.** **:. ::. : . .
