# Supplementary material for: Immune evasion activities of accessory proteins Vpu, Nef and Vif are conserved in acute and chronic HIV-1 infection
Source: Virology. 2015 Aug;482:72–8. doi: 10.1016/j.virol.2015.03.015 (PMC4503796; doi:10.1016/j.virol.2015.03.015)
Supplement: Supplementary file 2 — Supplementary Figure 2: amino acid alignment of Vif alleles used in this study [file mmc2.doc]

ZM247 MENRWQVLIVWQVDRMRIRTWNSLVKHHMYVSRRANGWFYRHHYETRHPKISSEVHIPLG-DARLVIKTYWGLHTGERDWHLGHGVSIEWRLRRYSTQVD

ZM249 MENRWQVLIVWQVDRMRIRTWNSLVKHHMYVSRRANGWFYRHHYESRHPKISSEVHIPLG-EARLVIKTYWGLHTGEREWHLGHGVSIEWRLRRFSTQVD

ZM246F MENRWQVLIVWQVDRMRIRTWNSLVKHHMYVSKRTNGWFYRHHYESRHPRVSSEVHIPLG-EARLVIRTYWGLHTGEREWHLGNGVSVEWRLRKYSTQID

CH432 MENRWQVLIVWQVDRMRIKTWNSLVKHHMYVSKRAKGWFYRHHFESRNPKVSSEVHIPLG-EARLVIKTYWGLQTGERDWHLGNGVSIEWRLRRYSTQVD

CH457 MENRWQVLIVWQIDRMRIRTWNSLVKHHMYVSRRANGWYYRHHYESRHPKVSSEVHIPLG-DARLVIKTYWGLQTGEREWHLGHGVSIEWRLRRYSTQVD

CH534 MENRWQVLIVWQIDRMKIRTWNSLVKHHMYISKKASGWFYKHHYESRHPKVSSEVHIPLG-DAKLVVKTYWGLQTGERAWHLGHGASIEWRLREYSTQVD

CH40 MENRWQVMIVWQVDRMRIRAWKSLVKHHMYVSGRARGWFYRHHYESTHPRISSEVHIPLG-EDRLVVTTYWGLHTGERNWHLGQGVSIEWRKKRYSTQVD

CH77 MENRWQVMIVWQVDRMRINTWKSLVKHHMYVSRKARRWFYRHHYESTHPRISSEVHIPLGDEAKLVIITYWGLHTGERDWHLGQGVSIEWRQKRYSTQVD

WITO MENRWQVMIVWQVDRMRIRAWNSLVKHHMYISKKAAGWFYRHHYESQHPKISSEVHIPIG-EARLVITTYWGLNTGERDWHLGQGVSIEWRKKKYSTQVD

STCOr1 MENRWQVMIVWQVDRMRIRTWKSLVKHHMYISKKARRWFYRHHYESTHPRISSEVHIPLG-DAKLVITTYWGLHTGEREWHLGQGVSIEWRQRRYNTQLT

RHGA MENRWQVMIVWQVDRMRIRTWKSLVKHHMYTSKKAKGWFYRHHYESTHPRISSEVHIPLG-DARLVITTYWGLHTGEREWHLGQGVSIEWRKKRYSTQVD

WARO MENRWQVMIVWQVDRMRIRAWKSLVKHHMYVSKKAQGWFYRHHYDSRHPRISSEVHIPLG-EAKLVVTTYWGLNTGERDWHLGQGVSIEWRKRRYSTQVD

*******:****:***.*.:*:******** * .: *:*.**:.: :*. :*******:* . .**: *****:**** ****:*.*:*** ..:.**:

ZM247 PGLADQLIHMHYFDCFADSAIRKAILGHIVIPRCDYQAGHNKVGSLQYLALTALIKPKKIKPPLPSVGKLVEDRWNKPQKTKGRKGNHTMNGH

ZM246F PGLADQLIHMHYFDCFADSAIRKAILGHIVSPRCDYQAGHNKVGSLQYLALTALIKPKKVKPPLPSVKKLVEDRWNKPQKTRGRRGNHTMNGH

ZM249 PGLADQLIHMHYFDCFADSAIRKAILGQIVSPKCDYQAGHNKVGSLQYLALTALIKPKKIKPPLPSVQKLVEDRWNKPQKTRGHRGSHTMSGH

CH432 PGLADQLIHMHYFDCFADSAIRQAILGHIVIPRCDYQAGHNKVGSLQYLALTALIKPRKRKPPLPSVRKLAEDRWNNPQKTRGRRGNHTMNGH

CH457 PGLADQLIHMHYFDCFADSAIRKAILGHIVTPRCDYQAGHNQVGSLQYLALTALVKPKKIKPPLPSVRKLVEDRWNNPQKTRGRRGNHIMNGH

CH534 PGLADQLIHMHYFDCFADSAIRKALLGHIVSPRCEYQAGHNKVGSLQYLALTALIKPKKTKPPLPSVSKLVEDRWNKPQKTRGRRGNHTMNGH

CH40 PNLADQLIHLYYFDCFSESAIRKAILGRIVSPRCDYQAGHNKVGSLQYLALTALIKPKRTKPPLPSVTKLTEDRWNKPQKTKGHRGSHTMNGH

CH77 PNLADQLIHLYYFDCFSESAIRNAILGHIVSPRCEYQAGHNKIGSLQYLALTALIKPKRRKPPLPSVAKLTEDRWNKPQKTKDHRGSHTMNGH

WITO PDLADQLIHLYYFDCFSESAIRNTILGHRVSPRCEYQAGHNKVGSLQYLALTALIKPKKRKPPLPSVAKLTEDRWNKPQRTKGRRGSHTMNGH

STCOr1 PDLADQLIHLYYFDCFSESAIRNAILGRIVSPSCEYQAGHNKVGSLQYLALAALIKSKRIKPPLPSVTKLTEDRWNKPQKTKGHRGSHTLNGH

RHGA PKLADHLIHLHYFDCFSESAIRNAILGHIVSPSCEYQAGHNKVGSLQYLALAALISPKKRKPPLPSVAKLTEDRWNKPQKIKGHRGSHTMNGH

WARO PNLADQLIHLYYFDCFSESAIRNALLGQIVRPKCAYQAGHNKVGSLQYLALVALTTPKKIKPPLPSVAKLTEDRWNKPQKTKGHRGSHTMNGH

* ***:***::*****:.****:::**. * * * ******::********.** .... ******* **.*****:**. ....*.* :.**
