# Supplementary material for: Immune evasion activities of accessory proteins Vpu, Nef and Vif are conserved in acute and chronic HIV-1 infection
Source: Virology. 2015 Aug;482:72–8. doi: 10.1016/j.virol.2015.03.015 (PMC4503796; doi:10.1016/j.virol.2015.03.015)
Supplement: Supplementary file 3 — Supplementary Figure 3: amino acid alignment of Nef alleles used in this study [file mmc3.doc]

ZM246F MGGKWSKSSPVGWPNIRERIRRT---------DPAAEG-------VGTASQDLDRYGALTTSNTATNNPACAWLEAQEEEE--EVGFPVRPQVPLRPMTY

ZM247v1 MGGKWSKSSVVGWPAVRERLRKT---------EPAAEG-------VGAASQDLDKHGALTTSNTARNNADVAWLEAQEEEG--EVGFPVRPQVPLRPMTY

ZM249 MGGKWSKSSIVGWPAVRERIRRTNPM-----TERAAAG-------VGAVSQDLDRFGAVTSSNTAATNADVACLEAQEEEG--EVGFPVRPQVPLRPMTY

CH432 MGGKWSKSKRSDWPVVRERIRQT---------EPAAEG-------VGAASQDLGKHGAITSSNTDTNNADCAWLRAQEEED--DVGFPVRPQVPLRPMTY

CH457 MGGKWSKCSMVGWPEVRERIRRT---------APAAEG-------VGAASRDLDRHGAITSSNTATTNAACAWLEAQEEAE--EVGFPVRPQVPLRPMTY

CH534 MGGKWSKSSIVGWPEVRERIRRT---------RPAAEGEKTAAEGVGTASQDLDKHGALTTSNTARNNDACAWLEAQEEDG--DVGFPVRPQVPLRPMTF

CH40 MGGKWSKCSVVGWPSVRERMRR---------AEPAAEG-------VGAVSRDLEKHGAITSSNTAATNADCAWLEAQEEG---EVGFPVRPQVPLRPMTF

CH77 MGGKWSK--FAGWPAVRERMRRAGARERRRRDEPAAVG-------VGPASQDLAKHGAITSSNTVSNNADCAWLEAQEEEE--EVGFPVRPQVPVRPMTY

WITO MGGKWSKSWKIGWPTVRERMRRAEP-------EPAAVG-------VGAVSRDLERHGAVTSSNTATNNADSAWLEAQAQEEDNEVGFPVRPQVPVRPMTY

STCOr1 MGGKWSKRSVPGWSVIRERMRQTEPKMRQ--TEPAATG-------VGAASRDLERHGALTVNNTAENNADCAWVEAQEEEG--EVGFPVKPQVPLRPMTY

RHGA MGNKWSRSSVVGWPAIRERMQR---------TEPAAVG-------VGAVSRDLEKHGAITSSNTAATNADCAWLEAQEEE---EVGFPVRPQVPLRPMTY

WARO MGNKWSKSSLVGWPKIRERMRQ---------TEPAADG-------VGAASRDLEKHGAITSSNTATNNAACAWLEAQEDE---DVGFPVRPQVPLRPMTY

**.***. .*. :***:.. ** * **..*.** ..**:* .** .* * :.** . .*****.****:****:

ZM246F KGAFDLSFFLKEKGGLEGLIYSKKRQDILDLWVHNTQGFFPDWQCYTPGPGVRYPLTFGWCFKLVPVDPKEVEEANEGENNCLLHPMSLHGMEDADREVL

ZM247v1 KGAVDLSFFLKEKGGLEGLIYSKKRQEILDLWVYHTQGFFPDWQNYTPGPGIRYPLTFGWCFKLVPVDPREVEEANEGENNCLLHPMSLHGVEDECREVL

ZM249 KSAVDLSFFLKEKGGLDGLVYSKKRQDILDLWVYNTQGFFPDWQNYTPGPGIRYPLTFGWCYKLVPVDPREVEEANEGENLSLLHPMCQHGIEDTEREVL

CH432 KEAFDLSFFLKEKGGLEGLIYSKKRQEILDLWVYHTQGFFPDWQNYTPGPGVRYPLTFGWCYKLVPVDPSEVEEASGGEDNCLLHPMNLHGIEDEHREVL

CH457 KGAVDLSFFLKEKGGLEGLIYSKKRQDILDLWVYHTQGYFPDWQNYTPGPGVRYPLTLGWCYKLVPVDPKEVEEETEGENNSLLHPMSLHGMEDEHREVL

CH534 KAAFDLSFFLKEKGGLEGLIYSRKRQEILDLWVHNTQGFFPDWQNYTPGPGVRYPLTFGWCFKLVPVDPREVEEANEGENNCLLHPMSQHGMEDEDREVL

CH40 KGALDLSHFLKEKGGLEGLIYSQKRQDILDLWVYHTQGYFPDWQNYTPGPGTRFPLTFGWCFKLVPVDPGKVEEANKGENNCLLHPMSQHGMDDPEREVL

CH77 KAALDLSHFLKEKGGLEGLIYSQQRKDILDLWVYNTQGFFPDWQNYTPGPGPRFPLTFGWCFKLVPVEPEEVEKANEGENNCLLHPMSQHGTDDPEKEVL

WITO KAAVDLSHFLKEKGGLDGLIYSQQRQDILDLWVYNTQGFFPDWQNYTPGPGTRYPLTFGWCYKLVPVEPEEVEKANEGENNSLLHPMGLHGMDDPEKEVL

STCOr1 KGALDLSHFLKEKGGLEGLVWSQKRQDILDLWVYNTQGFFPDWHNYTPGPGPRFPLTFGWCFKLVPVTPEEVEAANEGENNSLLHPMSLHGMEDPEGEVL

RHGA KGALDLSHFLREKGGLEGLVHSQKRQDILDLWVYNTQGYFPDWQNYTPGPGVRWPLTFGWCFKLVPVEPEKIEEANKGENNCLLHPMSQHGMDDPEREVL

WARO KAAVDLSHFLKEKGGLEGLIHSQKRQDILDLWMYHTQGYFPDWQNYTPGPGVRYPLTFGWCFKLVPVDPEKIEEATEGEDNKLLHPMCLHGMEDPEKEVL

* *.***.**.*****.**: *.:*:.*****:::***:****: ****** *:***:***:***** * ::* . **. ***** ** .* ***

ZM246F MWKFDSSLAHRHMARELHPEYYKDC

ZM247v1 KWKFDSHLARRHMARELHPEFYKDC

ZM249 IWKFDSHLARRHMAREIHPEYYKDC

CH432 KWKFDSQLARRHLAREKHPEFYKDC

CH457 KWKFDSMLARRHMAREKHPEYYKDC

CH534 MWKFDISLAHRHMAREIHPEYYKDC

CH40 VWRFDSSLAFRHVARELHPEYYKNC

CH77 AWRFDSRLAFQHVAREIHPEFYKDC

WITO MWKFDSRLAFHHMAREKHPEFYKDC

STCOr1 VWKFDSRLAVHHVAKVLHPEYYKNC

RHGA VWKFDSHLAVHHVAREKHPEYYENC

WARO VWKFDSSLALHHVARELHPEYYKNC

*.** ** .*:*. ***:*:.*
